# High Dynamic Pixel Structure Based on an Adaptive Integrating Capacitor †

**DOI:** 10.3390/s23229071

**Published:** 2023-11-09

**Authors:** Suiyang Liu, Zhongjie Guo, Ruiming Xu, Ningmei Yu

**Affiliations:** Department of Electronic Engineering, Xi’an University of Technology, No. 5, Jinhua South Road, Xi’an 710048, China; syliu@stu.xaut.edu.cn (S.L.); rmxu@stu.xaut.edu.cn (R.X.); yunm@xaut.edu.cn (N.Y.)

**Keywords:** CMOS infrared image sensor, adaptive capacitance, high dynamic range, large full well capacity

## Abstract

Infrared image sensing technology has received widespread attention due to its advantages of not being affected by the environment, good target recognition, and high anti-interference ability. However, with the improvement of the integration of the infrared focal plane, the dynamic range of the photoelectric system is difficult to improve, that is, the restrictive trade-off between noise and full well capacity is particularly prominent. Since the capacitance of the inversion MOS capacitor changes with the gate–source voltage adaptively, the inversion MOS capacitor is used as the capacitor in the infrared pixel circuit, which can solve the contradiction between noise in low light and full well capacity in high light. To this end, a highly dynamic pixel structure based on adaptive capacitance is proposed, so that the capacitance of the infrared image sensor can automatically change from 6.5 fF to 37.5 fF as the light intensity increases. And based on 55 nm CMOS process technology, the performance parameters of an infrared image sensor with a 12,288 × 12,288 pixel array are studied. The research results show that a small-size pixel of 5.5 µm × 5.5 µm has a large full well capacity of 1.31 Me^−^ and a variable conversion gain, with a noise of less than 0.43 e^−^ and a dynamic range of more than 130 dB.

## 1. Introduction

CMOS image sensors have replaced CCDs as the mainstream since the mid-20th century, and are widely used in smartphones, surveillance cameras, autonomous driving, and artificial intelligence [1,2,3]. However, the light range detected by the general infrared CMOS image sensor is small, and physical information identification in low-lighting and high-lighting environments cannot be carried at the same time. It has a low dynamic range (DR), which affects the output image quality of the infrared image sensor. The images obtained by a high-dynamic image sensor can retain more detailed information, so its related research is essential. High-dynamic infrared image sensors can be realized by improving the system and the readout circuit, such as building a high-dynamic image data acquisition system or multiple captures readout circuit, to achieve high-dynamic imaging or a combination of multiple images [4,5,6,7]. However, the superposition of multiple images leads to the continuous superposition of noise. The high-dynamic infrared pixel improves the pixel structure. A single imaging avoids the shortcomings of noise superposition and has the advantage of simple structure [8].

The core of CMOS infrared image sensors lies in pixels, so researchers have proposed solutions with different advantages for the problem of low DR [9]. 

Zaitsu uses 3D stacking technology to increase capacitance, full well capacity (FWC), and DR [10]. Maasa also proposed to use a transverse overflow capacitor in each pixel. The capacitance density of this capacitor is 24.3 fF/µm^2^, which is about 5 times that of the planar MOS capacitor [11]. It can achieve a very high FWC of 24.3 Me^−^ and the *DR* to 130 dB [12]. Uchida has developed a photo-diode structure that increases the *DR* to 97 dB [13]. However, all three methods have the disadvantages of complicated processes and difficulty improving the yield [14,15].

Yi Zhuo proposed to reduce noise by integrating correlated double sampling (CDS) technology in the pixel, increasing the *DR* from 60 dB to 74 dB [16], but the internally integrated CDS will take up additional area. Qian proposed a method for subtracting the internal charge of the pixel, which not only improves the signal-to-noise ratio of the dim signal, but also avoids the saturation of the bright signal, and increases the *DR* to 74 dB [17]. However, there are comparators and subtractors inside the pixel, which take up too much area [18]. Ko uses the characteristics of linear pixels with high sensitivity at low illumination and logarithmic pixels with high sensitivity at high illumination to propose a linear logarithmic active pixel structure, which simultaneously obtains the *DR* of linear pixel and logarithmic pixel, achieving a high *DR* of 152 dB [19]. However, the use of two sets of pixel units will take up more area. In short, these three methods will affect the fill factor of the pixel [20].

Kuznetsov proposed to adaptively adjust the integration time in each unit of the readout circuit to ensure linearity and conversion gain (CG) under moderate light, but reduce the sensitivity when approaching the FWC. This method can increase the *DR* to 100 dB. However, this method of adjusting the integration time will bring a lot of trouble to the timing arrangement and the design of digital circuits [21].

In order to achieve a high DR image sensor that combines with existing processes without affecting the fill factor and simple timing, based on the definition of DR, a mathematical model is established to analyze the restriction trade-off between the noise and the FWC of the infrared image sensor. According to the trade-off between the capacitance value of the adaptive capacitor and the light intensity, the pixel structure of the adaptive capacitor is proposed, which is very sensitive to light changes in low light, but it is not easy to reach the FWC in high light. At the same time, a 12,288 × 12,288 large infrared pixel array has been used to study linearity, CG, FWC, and noise, and, ultimately, improve the *DR*.

**Figure 1:**
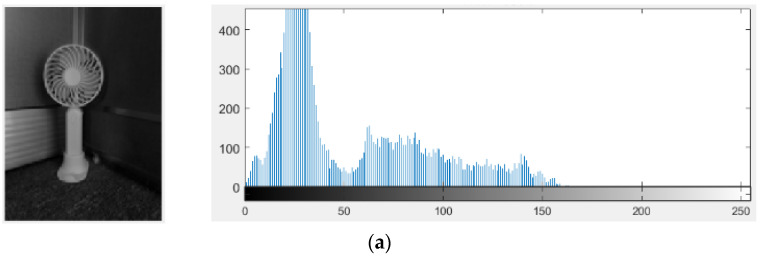

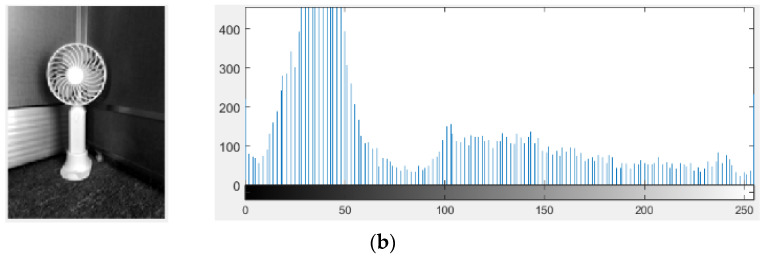
(**a**) Original image and PCH. (**b**) Output image and PCH of the high DR image sensor.

To intuitively display the imaging effect of the high DR pixel proposed in this paper, MATLAB is used to simulate the output image and photon-counting histogram (PCH) of the high DR image sensor, as shown in Figure 1. Figure 1a shows the original image and its PCH. Figure 1b shows the image and its PCH after being captured by this high DR image sensor. It can be seen that the image quality is obviously improved, and the *DR* is extended under both high-light and low-light conditions.

## 2. Analysis of the Restriction Trade-Off between Noise and FWC

The *DR* of an infrared image sensor is expressed as Equation (1) [22]

(1) $$DR=20\log_{10}\frac{Q_{FWC}}{Q_{noise}}\;$$

where *Q_FWC_* is the number of electrons that an integrating capacitor can hold when it reaches full well and *Q_noise_* is the equivalent number of noise electrons of the image sensor. As shown in Equation (1), increasing the *DR* of the infrared image sensor requires increasing the FWC while reducing the noise.

The FWC of the infrared image sensor can be expressed according to Equation (2) [23]

(2) $$\;Q_{FWC}=\frac{1}{q}\int C_{int}VdV\;$$

where q is the electronic charge, *C_int_* is the capacitance value of the capacitor, and *V* is the swing of the infrared image sensor.

Equation (3) is the *Q_noise_* of the image sensor, including all the noise generated inside the pixel, which the main part is the reset noise and shot noise during the readout process

(3) $$\;Q_{noise}=\frac{C_{int}V_{noise}}{q}\;$$

where *V_noise_* is the noise voltage. Since there is a programmable gain amplifier in the post-stage readout circuit, the noise generated by the readout circuit can be ignored when converted to pixels.

From Equations (2) and (3), it can be seen that increasing the FWC requires increasing the capacitance value while decreasing the noise requires decreasing the capacitance value. Therefore, for a high-dynamic infrared image sensor, there is a restrictive trade-off between noise and FWC.

## 3. Adaptive Capacitor Design

Due to the restrictive trade-off between FWC and noise for a high-dynamic infrared image sensor, an adaptive capacitor with a larger capacitance value in high light and a smaller capacitance value in low light will meet the needs of the high-dynamic infrared image sensor. Since there is a natural correspondence between the gate–source voltage (*V_gs_*) of a MOS capacitor and the capacitance value, a MOS capacitor is used as an adaptive integrating capacitor for highly dynamic pixels.

**Figure 2:**
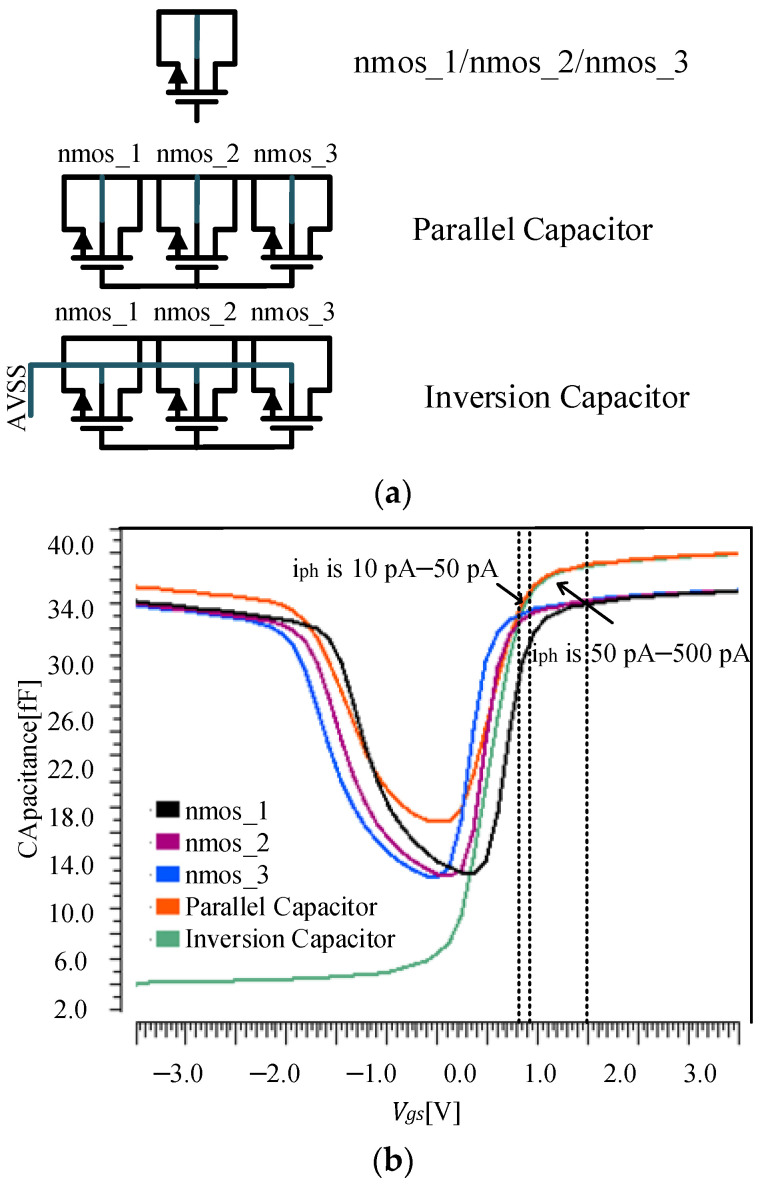
(**a**) Structure and (**b**) capacitance value versus *V_gs_* curve of adaptive integrating capacitors.

As shown in Figure 2a, three MOS capacitors with width and length of 3 µm are used, and the MOSFET types are nmos_1, nmos_2, and nmos_3, respectively. The three capacitors have the same thickness of gate oxide layer, all of which are 7.03 nm, and different threshold voltages, which are 0.62 V, 0.4 V, and 0.3 V, respectively. The simulation is carried out at 25 °C and the tt process corner. MOS capacitance values with *V_gs_* are shown as black, purple, and blue curves in Figure 2b. It can be seen that the capacitance values of these three types of capacitors are similar in the strong inversion and accumulation regions. In the inversion region with variable capacitance values, the curves of capacitance values versus *V_gs_* for all three types of capacitors have steep slopes, and the *V_gs_* region with variable capacitance values is very small. Therefore, they cannot be used directly as adaptive integrating capacitors for highly dynamic pixels.

To increase the voltage range over which the capacitance value of the MOS capacitor is variable, the nmos_1, nmos_2, and nmos_3 capacitors with an area of 1 µm × 3 µm are connected in parallel to obtain a parallel capacitor with a total area of 9 µm^2^ as shown in Figure 2a. The simulated capacitance value of the parallel capacitor varies with *V_gs_* at 25 °C and tt process corner, as shown by the orange curve in Figure 2b. It can be seen that the capacitance value of the parallel capacitor varies more gently with *V_gs_* in the inversion region where *V_gs_* is from 0 V to 1.5 V, and its capacitance value is larger than that of a single type of MOS capacitor.

The inversion MOS capacitor is obtained by grounding the substrate of the parallel capacitor as shown in Figure 2a, and its capacitance value varies with *V_gs_* as shown by the green curve in Figure 2b. The capacitance value of the inversion MOS capacitor is only 6.5 fF when *V_gs_* is 0 V. The capacitance value of the inversion MOS capacitor is 37.5 fF when *V_gs_* is 1.5 V. It can be seen that it has the advantages of a large area of *V_gs_* with variable capacitance value and a large change in capacitance value.

## 4. Adaptive Capacitor Infrared Image Sensor Structure

To study the real effect of inversion MOS capacitors for high-dynamic infrared image sensors, the 55 nm 1P4M CIS process platform was used to build a 12,288 × 12,288 pixel array infrared image sensor structure based on an adaptive capacitor.

**Figure 3:**
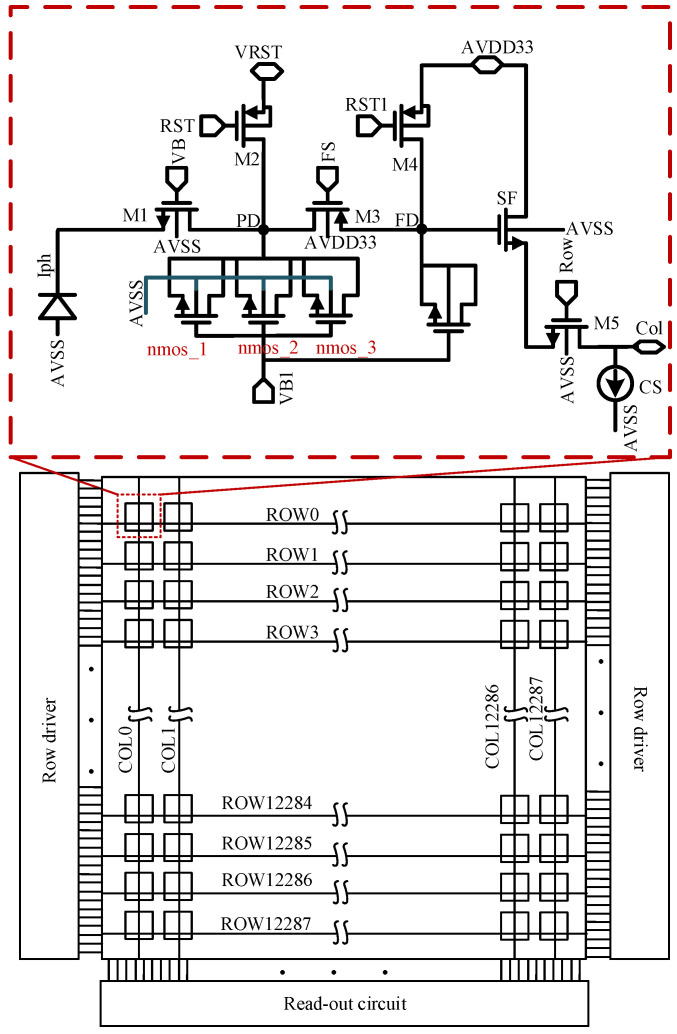
The basic architecture of a CMOS infrared image sensor based on adapting capacitance.

The structure of the adaptive capacitor infrared image sensor is shown in Figure 3. The red box in Figure 3 indicates the design of the adaptive capacitor pixel structure. The pixel adopts a 5-transistor (5T) structure. The innovation is that the value of integration capacitance can be adjusted adaptively according to the photo-current, thereby improving the *DR* of the pixel.

As shown in Figure 3, the adaptive integration capacitor infrared image sensor mainly includes three parts: a 12,288 × 12,288 pixel array, a row driver circuit, and a readout circuit. The row driver circuit is used to transmit the global reset signal (RST), electric charge transfer control signal (FS), row reset signal (RST1), and row selection control signal (Row). The pixel unit is 5T pixels [24], including an electric charge injection transistor (M1), a global reset transistor (M2), a row selection control transistor (M5), and a source follower (SF). In addition, it also includes a photo-diode (Iph), an electric charge transfer transistor (M3), a row reset transistor (M4), and an adaptive capacitor. Define the integration node as PD and the storage node as FD node. To achieve separate testing of the pixel unit, the reset supply and the source follower supply are separated.

During the integration time (*t_int_*), *V_PD_* gradually decreases from the reset voltage V_RST_ as the photo-current (*i_ph_*) is injected, as shown in Equation (4)

(4) $$\;V_{PD}=V_{\mathrm{RST}}-\frac{i_{ph}t_{int}}{C_{int}}\;$$

The voltage *V_gs_* across the capacitor is the difference between the gate voltage (V_VB1_) and the source voltage (*V_PD_*). According to Equation (4), Equation (5) is obtained.

(5) $$\;V_{gs}=V_{\mathrm{VB}1}-V_{\mathrm{RST}}+\frac{i_{ph}t_{int}}{C_{int}}\;$$

V_VB1_ is a bias voltage and V_RST_ is the reset voltage in Figure 3, both set to 3.3 V. The relationship between *V_gs_* and *C_int_* is shown in Equation (6).

(6) $$\;V_{gs}=\frac{i_{ph}t_{int}}{C_{int}}\;$$

According to the above analysis, for the high-dynamic pixel circuit in Figure 3, RST is turned on and resets the source and drain of the MOS capacitor to 3.3 V before the pixel is integrated, *V_gs_* is 0 V. According to Equation (4), *V_PD_* gradually decreases as the integration progresses. According to Equation (6), *V_gs_* gradually increases. Corresponding to the change of *V_gs_* in Figure 2, with the increase in integrating electric charge, *V_gs_* gradually increases from 0 V to 2.4 V, and the MOS capacitor works in the inversion region. The maximum value of *V_gs_* is shown in Equation (7)

(7) $$\begin{array}{c} {\;V}_{\mathrm{gs},\max}=V_{\mathrm{VB}1}-V_{\mathrm{PD},\min}\\ =V_{\mathrm{VB}1}-\left(V_{\mathrm{ta}}+V_{\mathrm{th}}\right)\\ =3.3\;V-\left(0.2+0.7\right)\;V=2.4\;V\; \end{array}$$

V_ta_ is the voltage across the tail current source (CS) in Figure 3, which is measured to be 0.2 V in the current process. V_th_ is the threshold voltage of the SF, and it also is the smallest voltage that can be output by the SF, which is measured to be 0.7 V in the current process.

As Equation (7), in high light, the maximum *V_gs_* can be increased to 2.4 V, while in ideal dark light, the minimum *V_gs_* can be reduced to 0 V. Corresponding to the curve in Figure 2, the capacitance value in high light is 5.4 times that in dark light so that the adaptive capacitance infrared pixel has a larger FWC in high light and lower noise in dark light. The infrared image sensor will achieve a greater dynamic range.

**Figure 4:**
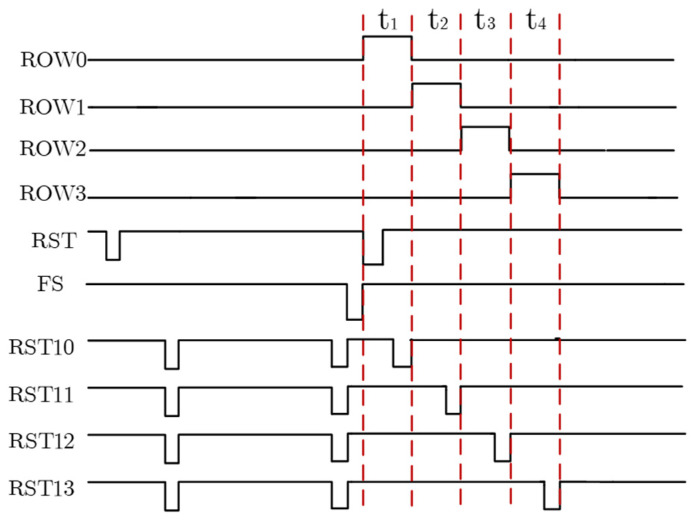
The timing diagram of pixels.

Figure 4 shows the timing of the first four-row row-driver circuits. VB is a fixed bias of 1 V. The photo-electron collected in the photo-diode is injected into the integrating capacitor with low power consumption through an electric charge transfer transistor operating in the sub-threshold region. At this time, the current I_DS_ and *V_gs_* have an exponential relationship, as shown in Equation (8)

(8) $$\;I_{DS}=I_{0}\exp\left(\frac{qV_{gs}}{nkT}\right)$$

*I*_0_ represents the saturation current, and its value is proportional to *W*/*L*; *n* > 1 is a non-ideal factor. According to Equation (8), *I_DS_* has nothing to do with source–drain voltage at this time. Therefore, as integration progresses, the voltage on the integrating capacitor will change greatly, but it will not affect the bias voltage of the photo-diode.

As can be seen from Figure 4, in the reset stage, RST is used as the global reset signal, which is low level first, resetting the integrating capacitor node PD. Then, the RST10-RST13 are low level at the same time to reset the transferring capacitor node FD. Secondly, the signal FS is low level to transfer the charge. Finally, in the readout stage, ROW0-ROW3 are high-level one after another during t_1_-t_4_, indicating that the signal voltages are read out row by row. The column bus voltage of pixels drops and the signal voltages are displayed. After that, RST10-RST13 are low level row by row, the column bus voltage of the pixel increases, and the reset voltages are displayed. The post-stage circuit uses CDS technology to sample signal voltages and reset voltages.

## 5. Layout Design and Simulation Data Analysis

To study the imaging effect of a high-dynamic infrared image sensor based on the adaptive capacitor, on the 55 nm 1P4M CIS process platform, the layout design and simulation data analysis of the infrared image sensor are carried out.

**Figure 5:**
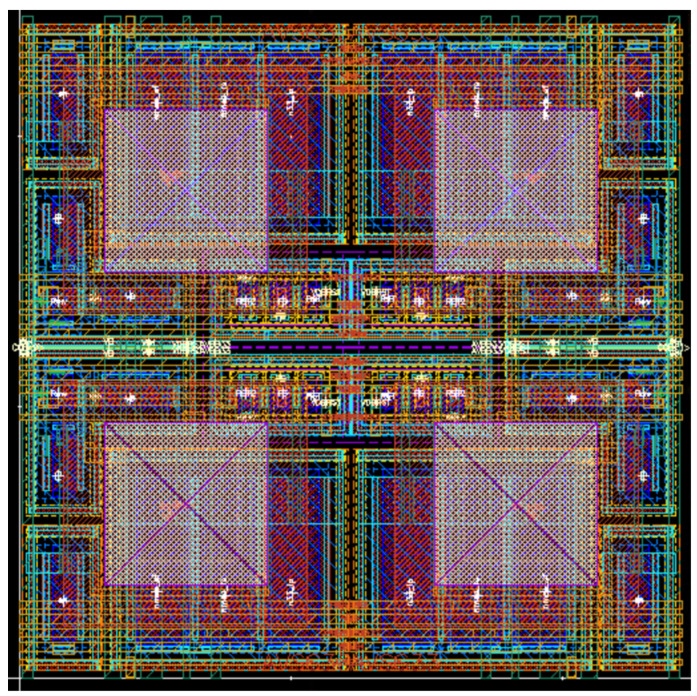
A 2 × 2 pixel array layout.

The layout design of four pixels is shown in Figure 5.

To compact the layout, a 2 × 2 pixel unit is used as the smallest repeating unit of the pixel array in the layout design, and the N-well is further shared. The pixel pitch is 5.5 µm. Figure 5 shows that the integrating capacitor uses different types of NMOS capacitors to be distributed on the upper and lower sides of the layout. The PMOS reset transistor is designed in the middle to share the N-well and achieve greater space utilization and pixel voltage swing.

Using an adaptive capacitor does not occupy more chip area or affect the pixel fill factor. Because this pixel structure does not increase the number of MOSFET compared with the general 5T structure, only the type of MOS capacitor is replaced. And the area occupied by different types of MOS capacitors is also equal. The structure proposed in this paper is a back-illuminated CMOS image sensor, as shown in Figure 5. The white square is a hole connected to a HgCdTe photo-diode for growing an indium column of the flip chip. Because the HgCdTe photo-diode is stacked on the pixel circuit, the fill factor can reach 100%.

In the 12,288 × 12,288 pixel array scale infrared image sensor, the influence of the adaptive capacitor on the noise, FWC, and DR are studied. Analog simulation was carried out at 25 °C and tt process corner. Under low light with a photo-current of 10–50 pA and high light conditions with a photo-current of 50–500 pA, the column signal of infrared pixels is simulated.

**Figure 6:**
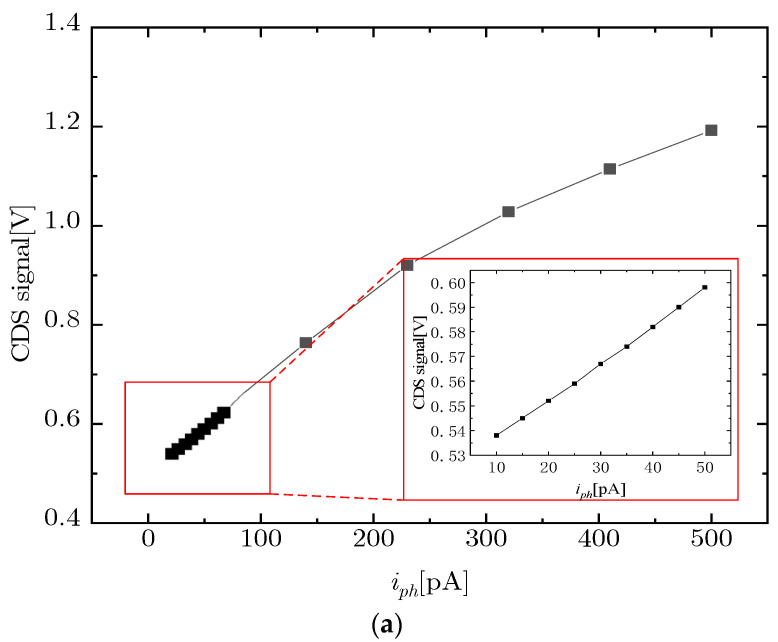

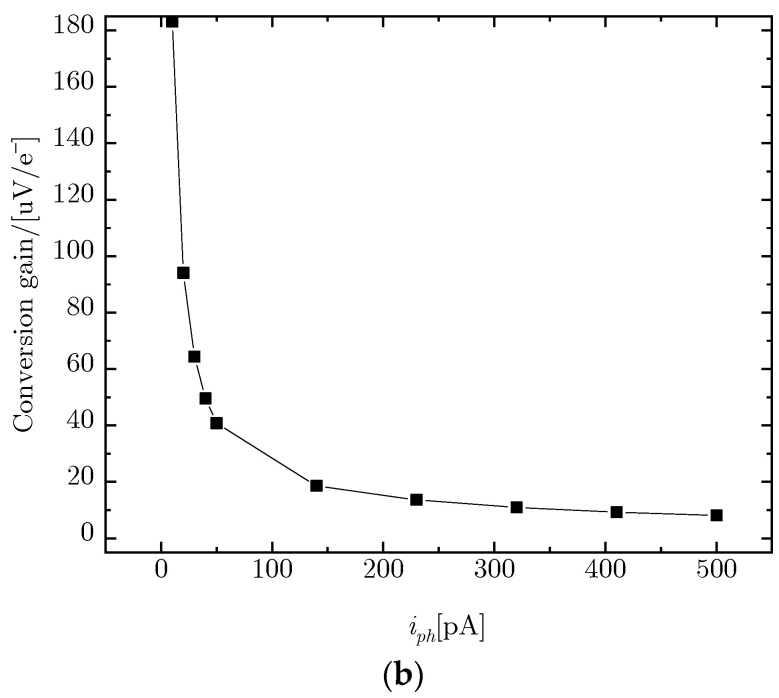
(**a**) CDS signal and (**b**) *CG* changed with photo-current.

Linearity is an important performance index of infrared image sensors. The linearity of the image sensor can be calculated by the curve of the CDS signal change with photo-current in Figure 6a. The CDS signal is obtained from the infrared pixel column signal, which is the difference between the reset voltage and the signal voltage. The output CDS signal less than 0.6 V is captured under low light, and the output CDS signal greater than 0.6 V is captured under high light.

As shown in Figure 2, the variation of *C_int_* has only one distinct inflection point near the threshold voltage over the range of *V_gs_* from 0 V to 2.4 V and therefore can be approximated to be segmentally linear. The relationship between *V_CDS_* and *C_int_* can be approximated and described as Equation (9)

(9) $$V_{CDS}=\{\begin{array}{c} a_{1}C_{int}\;\;\;\;\;\;\;\;0<V_{CDS}<V_{\mathrm{th}}\\ a_{2}C_{int}+b\;\;\;\;\;\;\;\;\;V_{CDS}>V_{\mathrm{th}} \end{array}\;$$

In this paper, the nonlinear correction circuit proposed in the literature [25] is used in the post-stage readout circuit so that the CDS signal is equal to the voltage across the variable integrating capacitor, *V_gs_* in Equation (6). Thus, Equation (6) can be written as Equation (10).

(10) $$Q_{in}=C_{int}V_{CDS}=i_{ph}t_{int}$$

The association with Equation (10) gives the relationship between *V_CDS_* and *i_ph_*, as in Equation (11)

(11) $$\{\begin{array}{c} V_{CDS}^{2}=a_{1}i_{ph}t_{int}\;\;\;\;\;\;\;\;\;\;\;\;\;\;\;\;\;\;\;\;0<V_{CDS}<V_{\mathrm{th}}\\ V_{CDS}\left(V_{CDS}-b\right)=a_{2}i_{ph}t_{int}\;\;\;\;\;\;\;\;\;V_{CDS}>V_{\mathrm{th}} \end{array}\;$$

It can be seen that *V_CDS_* is not strictly linear with *i_ph_*, but it can be approximated as such. This paper adopts a nonlinear correction circuit of replicating FD nodes proposed in the literature [25], which can perfectly solve the problem of non-linearity caused by the source follower. For the post-stage readout circuit, although the output of each stage of the readout circuit cannot be completely linear, the non-linearity of the post-stage readout circuit has been verified to be only 1%.

It is obtained that the CDS signal of the infrared pixel has good linearity in low and high light. Linearity was calculated as the maximum deviation of the CDS curve from the ideal straight line. The non-linearity is obtained by dividing the maximum deviation voltage by the voltage range of the CDS signal. Linearity is equal to 100% minus non-linearity. When the photo-current ranges from 10 pA to 50 pA, the linearity of the image sensor is calculated to be 96.7%. When the photo-current ranges from 50 pA to 500 pA, the linearity of the image sensor is calculated to be 86.6%. When the photo-current ranges from 10 pA to 500 pA, the linearity of the image sensor is calculated to be 86.5%.

Figure 2 shows that the capacitance changes with photo-current is 10–50 pA and 50–500 pA. According to Figure 2, in the area where the photo-current is 10–50 pA, the linearity of the capacitance change is good. Due to the substrate biasing effect of pixels, when photo-current in the range of 10–500 pA, the V_th_ changes from 1.1 V to 1 V. Owing to the V_th_, the change of the capacitance has an inflection point and is nonlinear, which affects the linearity of the image sensor.

Figure 6b also illustrates this problem. Although the *CG* in a photo-current of 10–50 pA has a high rate of change, the change is linear. The change rate of *CG* in 50–500 pA photo-current is low, but there is an inflection point at 140 pA, making the change in the *CG* nonlinear and affecting the image sensor’s linearity. This linearity problem is consistent with the traditional pixel structure and is widespread. Due to limited space, the paper focuses on solving the high dynamic problem. In the literature [25], the non-linearity problem has been analyzed and a solution has been proposed to eliminate it. 

*CG* is the ratio of the CDS signal to the number of photo-generated electrons [26]. The *CG* calculation equation is (12)

(12) $$CG=\frac{1\times q}{C_{int}}$$

where 1 represents an electron. Therefore, the effect of the adaptive integration capacitor on the linearity and sensitivity of the image sensor can be investigated by using the curve of the CDS signal and *CG*.

In low light and high light, the *CG* of the adaptive capacitor pixel is calculated by Equation (10), and its change curve with the photo-current is shown in Figure 6b. At low light, 10–50 pA photo-current, the *CG* drops rapidly from 183.1 µV/e^−^ to 40.8 µV/e^−^. The *CG* decreased slowly from 40.8 µV/e^−^ to 8.1 µV/e^−^ at high light with 50–500 pA photo-current. The transition region changed gently. It can be seen that in different lighting environments, the adaptive capacitor pixel has an adjustable *CG*. The adaptive capacitor infrared image sensor has excellent performance in low-light conditions.

The relationship between the signal-to-noise ratio of the infrared image sensor and the photo-current is Equation (13) [27].

(13) $$\;SNR=\frac{{\left(i_{ph}t_{int}\right)}^{2}}{qi_{ph}t_{int}+q^{2}\sigma_{Readout}^{2}},\;i_{ph}\leq \frac{qQ_{FWC}}{t_{int}}\;$$

**Figure 7:**
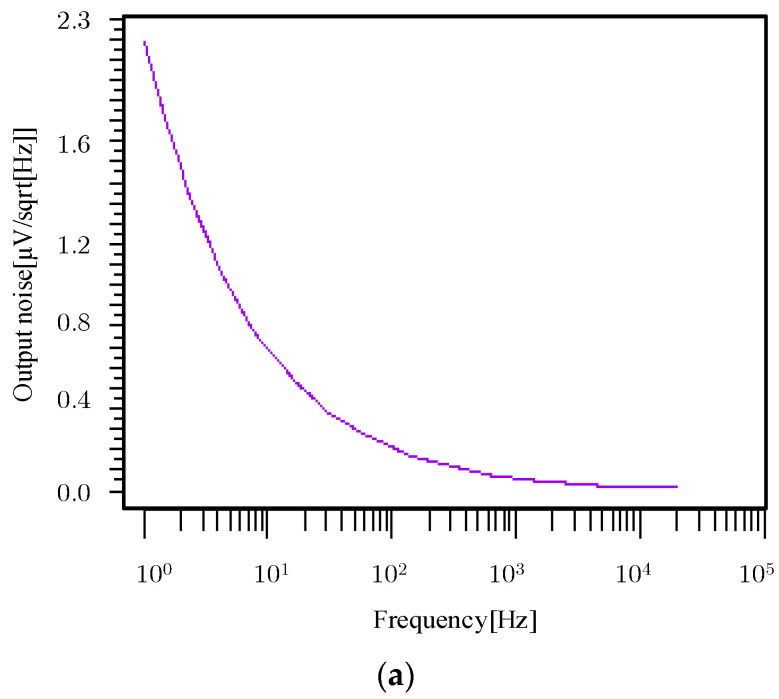

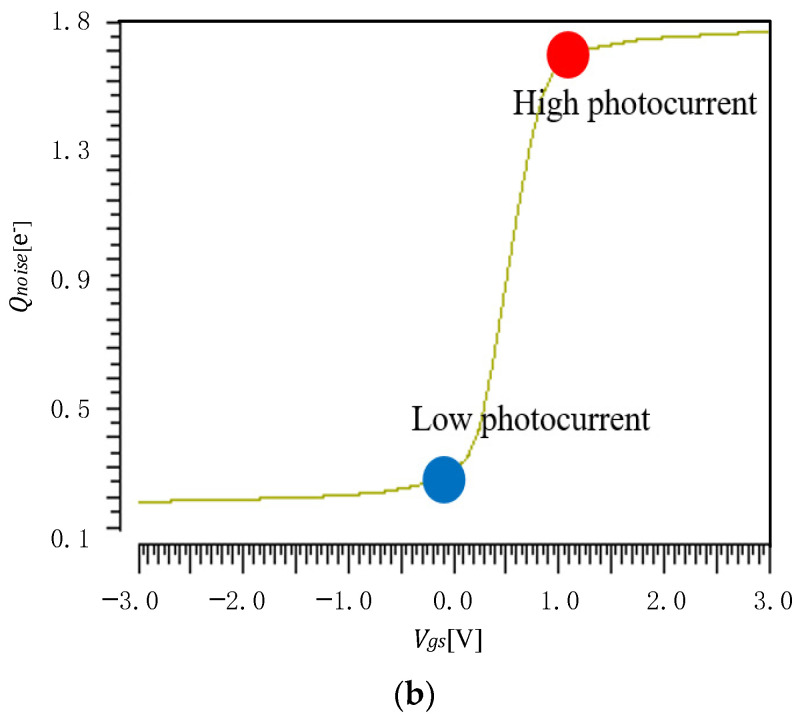
(**a**) The noise curve and (**b**) *Q_noise_* of 5T adaptive capacitor pixel.

Equation (13) represents the ratio of input signal power to average input reference noise power, where q*i_ph_t_int_* represents the shot noise generated by the optical signal, and modeled as a white Gaussian noise process; $\sigma_{Readout}^{2}$ is all the noise that affects the image sensor, and its types include fixed-pattern noise related to process variation, reset noise during readout, and 1/f noise-related temperature and channel length. The noise analysis of the adaptive capacitor pixel is shown in Figure 7.

The noise frequency domain curve of the pixel is shown in Figure 7a. The adaptive infrared pixel noise is mainly a low-frequency signal, such as flicker noise. The noise is 7.45 µV/Hz. According to Equation (3), due to the use of an adaptive capacitor, the equivalent number of noise electrons at different *V_gs_* changes with the change of capacitance value. The curve is shown in Figure 7b. According to Equation (6), the blue dot in the figure represents that under low light conditions, *V_gs_* is 0 V and the noise is only 0.3 e^−^. The red dot in the figure represents that under high light conditions, *V_gs_* is 1 V and the noise is 1.8 e^−^. It can be seen that the equivalent number of noise electrons increases with the increase in light intensity. It achieves excellent performance with low noise in low light.

**Figure 8:**
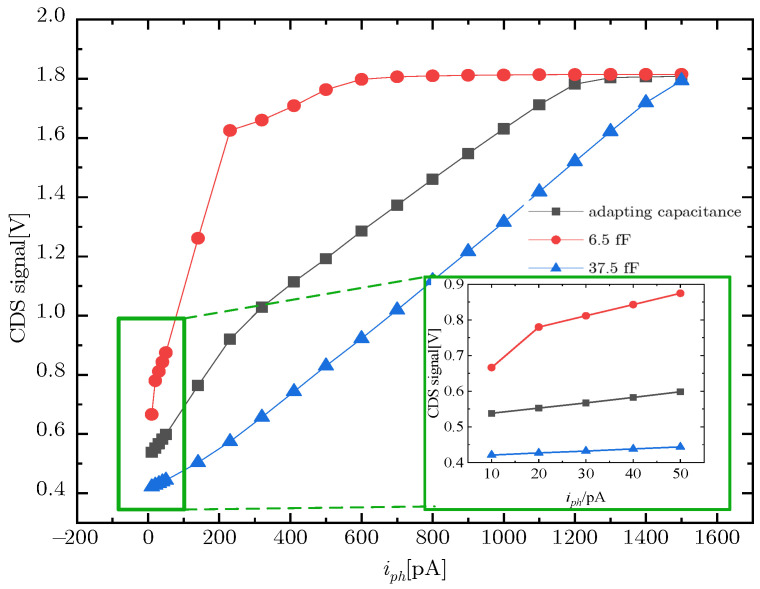
CDS signal curve of use adapting capacitor, 6.5 fF capacitor, 37.5 fF capacitor.

To study the change in capacitance value of the adaptive integrating capacitor under different light intensities, the pixel CDS signals using the adaptive integrating capacitor and a fixed capacitance value capacitor as the integrating capacitor is compared. The curve of CDS signal variation with photo-current is shown in Figure 8. The black curve in Figure 8 shows, an adaptive capacitor used as an integrating capacitor, CDS signal change with photo-current. For 5T infrared pixels with 6.5 fF and 37.5 fF fixed capacitors, the curve of the CDS signal is in red and blue, respectively.

It can be seen from Figure 8 that under low light conditions of 0–230 pA, the slope of the adaptive capacitor curve is closer to the red curve of 6.5 fF. Under the high light conditions of 230−1500 pA, the slope of the adaptive capacitor curve is closer to the blue curve of 37.5 fF. 

**Figure 9:**
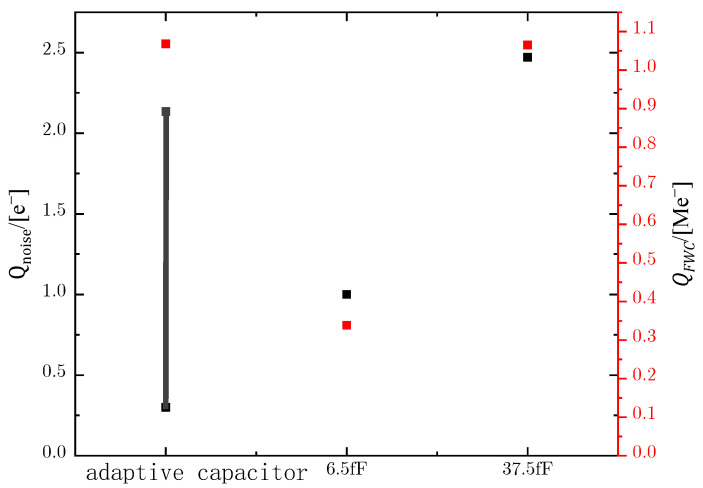
Noise and FWC of different capacitor 5T pixels.

To study the effectiveness of the adaptive integrating capacitor in reducing noise and increasing FWC, the pixel noise, and FWC with the adaptive capacitor are compared with those with a fixed capacitor. According to Equations (2) and (3), the noise and FWC of 5T pixels with different capacitors are shown in Figure 9.

Figure 9 shows that the adaptive capacitor pixel has less noise (0.3–1.8 e^−^) compared with the small capacitor of 6.5 fF, and a larger FWC (1.1 Me^−^) compared with the large capacitor of 37.5 fF. According to Equation (1) of *DR*, it can be seen that *DR* is determined by two factors, *Q_FWC_* and *Q_noise_*. As shown in Figure 9, the equivalent number of noise electrons of the image sensor with an adaptive capacitor is variable, so the *DR* calculated according to Equation (1) is 130–116 dB.

To further study the real performance of the adaptive capacitor image sensor, post-layout simulation is carried out based on a 55 nm CMOS process platform. The parameters used for post-layout simulation have been corrected based on measurements from actual sensors [27]. Under the process corner of 25 °C and tt, real performance parameters as shown in Table 1. Its various parameters are better than the pre-layout simulation under low light and high light conditions, and the consistency of pre-layout and post-layout simulation is achieved. Among them, the parasitic capacitor of the PD node is considered in the post-layout simulation of FWC. These parasitic capacitors can store additional charge, thereby increasing the FWC of the pixel circuit.

Since the device model files and parasitic parameter files called by the post-layout simulation have been corrected according to the measurements of the actual sensors, the performance parameters of the sensors proposed in this paper have the feasibility to be directly compared with other high-DR image sensors, as shown in Table 2. The pitch of the adaptive capacitor pixel is only 5.5 µm, which is much smaller than 16 µm of [11], 22 µm of [16], and 15 µm of [21], which is very advantageous. Compared with the highly DR method of increasing the subtractor in [12], increasing the counter in [16], or optimizing the structure of the photo-diode in [21], the adaptive capacitor design is simple. With a larger capacitor available area and FWC of 1.31 Me^−^. Although the *DR* of the literature [18] is large, the linearity is poor due to the use of logarithmic pixels. In comparison with the literature in Table 2, it is found that high DR image sensors with adaptive integrating capacitors not only have comparable *DR* but also have the advantage of using the existing process without affecting the fill factor and simplicity of timing.

## 6. Conclusions

In the process of the development of infrared image sensors to small size, large FWC, and low noise, high DR imaging has practical significance. Adaptive capacitors can be widely used in various pixel structures to achieve high DR imaging. Based on the 55 nm CMOS process platform, the research on an adaptive capacitor to improve the *DR* is carried out in a 12,288 × 12,288 ultra-large array infrared image sensor chip. Studies have shown that under low light signals with photo-current of 10–50 pA, the capacitance is close to 6.5 fF, the *CG* is as high as 39.2 µV/e^−^, and the noise is as low as 0.43 e^−^; when the photo-current is 50–500 pA for high light signals, the capacitance is close to 37.5 fF, the *CG* is reduced to 2.3 µV/e^−^, the FWC is increased to 1.31 Me^−^, and the *DR* can reach 130 dB. The adaptive capacitor solves the restriction trade-off between noise and FWC in pixel design. It provides certain theoretical guidance for the design of high-performance and large-array CMOS image sensors.

## Figures and Tables

**Table 1:** Post-layout simulation performance parameters of 5T infrared pixel circuit.

	Linearity (%)	*CG* (μV/e^−^)	*Q_noise_* (e^−^)	*DR* (dB)	*Q_FWC_* (Me^−^)
Low light (10–50 pA of photo-current)	97.5	110.6–24.8	0.4–2.2	130–116	1.31
High light (50–500 pA of photo-current)	96.6	24.8–6.5			

**Table 2:** Performance comparison of image sensors.

Reference	[11]	[12]	[16]	[18]	[21]	This Work
technology (nm)	180	-	65	180	-	55
pixel size (μm)	16 × 16	2.9 × 2.9	22 × 22	10.5 × 8.5	15 × 15	5.5 × 5.5
*Q_noise_* (e^−^)	7.6	0.78	61	-	6	0.43–2.2
*Q_FWC_* (Me^−^)	24.3	0.06	0.3	-	0.6	1.31
method	transverse overflow integrated grooved capacitor	new photo-diode structure	double exposure charge subtraction method	linear–logarithmic pixels	adaptive adjust the integration time	adaptive capacitor
*DR* (dB)	130	97	74	152	100	130–116

## Data Availability

The data presented in this study are available on request from the corresponding author. The data are not publicly available due to privacy.
